# Combined trochleoplasty and medial patellofemoral ligament reconstruction reduces patellar height

**DOI:** 10.1002/jeo2.70742

**Published:** 2026-05-06

**Authors:** Felix Riechelmann, Alexa Schaufler, Nadja Gasser, Paul Nardelli, Rohit Arora, Wolfgang Hackl

**Affiliations:** ^1^ Department of Orthopaedics and Trauma Surgery Medical University of Innsbruck Innsbruck Austria; ^2^ Medical University of Innsbruck Innsbruck Austria

**Keywords:** diagnostic imaging, MPFL reconstruction, patellar instability, patellofemoral Joint, tibial tubercle osteotomy, trochleoplasty

## Abstract

**Purpose:**

This study assessed whether trochleoplasty combined with medial patellofemoral ligament reconstruction (MPFLR) reduces patellar height, potentially obviating the need for distalizing tibial tubercle osteotomy (TTO).

**Methods:**

Patellar height was evaluated in 73 knees before and at a median of 6 months after patellar stabilisation using the Caton–Deschamps (CDI), Insall–Salvati (ISI) and Blackburne–Peel (BPI) indices.

**Results:**

MPFL reconstruction with trochleoplasty resulted in a sustained 8.1% postoperative reduction in the CDI (95% confidence interval [CI]: −14.1% to −2.1%; *p* < 0.001). The reduction was greater with MPFLR plus TTO (16.5%; −23.7% to −9.3%; *p* < 0.001) and maximal when MPFLR, trochleoplasty and TTO were performed together (22.9%; −33.1% to −12.6%; *p* < 0.001). ISI and BPI showed comparable reductions. While strong Pearson correlations among the indices (*r* = 0.6–0.9; all *p* < 0.001) were observed preoperatively, Bland–Altman analyses revealed a substantial bias of up to 25%. Inter‐index agreement for clinical classification (alta, normal, baja) was inconsistent, achieving at best a ‘moderate’ level (Weighted Cohen's kappa 0.36–0.60).

**Conclusion:**

Combined MPFL reconstruction and trochleoplasty reduce patellar height by approximately 10%. The interaction effect of various surgical procedures for PFI on patellar height, coupled with methodological inconsistencies in index‐based patellar height classification, challenges the validity of rigid cut‐off values for distalizing TTO.

**Level of Evidence:**

Level III.

**Abbreviations**
ANOVA: analysis of variance
AOSSM: American Orthopaedic Society for Sports Medicine
MPFL: medial patellofemoral ligament
MPFLR: medial patellofemoral ligament reconstruction
PFI: patellofemoral instability
TTO: tibial tubercle osteotomy
TT‐TG: tibial tubercle‐trochlear groove

## INTRODUCTION

Patellofemoral instability (PFI) is a multifactorial knee disorder often triggered by acute patellar dislocation with medial patellofemoral ligament (MPFL) rupture. It predominantly affects adolescents and young adults, especially females [18] and may lead to pain, functional impairment and progressive osteoarthritis [43]. Surgical stabilisation is indicated for recurrent dislocations, failed conservative therapy, or in the presence of anatomical risk factors like trochlear dysplasia, lateral maltracking or patella alta [6].

MPFL reconstruction (MPFLR) is the primary surgical treatment and is often sufficient as a standalone procedure [13, 24, 44]. In cases of high‐grade trochlear dysplasia, it is combined with trochleoplasty [3, 15, 32]. Lateral malalignment (TT–TG distance >20 mm) or patella alta (CDI > 1.2) may necessitate additional medialization or distalization via tibial tuberosity osteotomy (TTO) [22]. However, TTO is frequently associated with increased morbidity, including prolonged immobilisation, additional incisions and higher complication rates [36, 51, 54]. Patellar height is assessed via lateral knee radiographs using indices such as Caton–Deschamps index (CDI) [11], Insall–Salvati index (ISI) [25] and Blackburne–Peel index (BPI) [9]. These indices do not directly measure patella height; instead, they rely on the patella–tibia distance as a surrogate parameter [37, 52]. Elevated values correlate with higher dislocation risk and PFI prevalence [2, 15, 16, 30].

Surgical approaches are individualised based on anatomical risk profiles [14]. However, correcting one abnormality may normalise others, raising concerns about over‐treatment. We hypothesised that MPFL reconstruction and trochleoplasty alone would result in a significant postoperative reduction of radiographic patellar height indices, potentially eliminating the indication for concomitant TTO. Combined MPFLR + TTO and MPFLR+trochleoplasty + TTO were used as reference groups to quantify the relative magnitude of the effect. Furthermore, the degree of agreement between the CDI, ISI and BPI indices was assessed to evaluate their convergent validity in establishing the indication for TTO.

## METHODS

Patients who underwent surgery for PFI at the Department of Orthopaedics and Trauma Surgery, Medical University of Innsbruck, between 2010 and 2022, were retrospectively analysed. Patients were identified through the institutional clinical information system hosted on the Oracle Health Millennium Platform (Kansas City, MO).

### Screening and inclusion

Eligible patients were over 18 years of age, of any sex, and had undergone MPFL reconstruction in combination with either (a) trochleoplasty, (b) TTO or (c) combined trochleoplasty and TTO. Inclusion criteria required the availability of both preoperative and postoperative lateral knee radiographs. Patients with incomplete imaging records or those who underwent additional procedures such as rotational osteotomies were excluded. The study protocol was approved by the Ethics Committee of the Medical University of Innsbruck.

### Measurements

For each patient, two lateral knee radiographs were selected: the last image prior to surgery and the most recent available postoperative image. To ensure the reliability of patellar height measurements, all lateral radiographs were taken using a standardised source‐to‐image distance (SID) of 100 cm to ensure reproducible length measurements. All radiographs were screened for a true lateral projection at 30° knee flexion. Inclusion was contingent upon the superimposition of the posterior femoral condyles, a clear patellofemoral joint space, and neutral tibial rotation. Images failing these criteria were excluded and replaced by the chronologically nearest radiograph that met these quality standards. Measurements were performed using the Impax Viewer within the Picture Archiving and Communications System (Agfa Healthcare). The following parameters were recorded: distance from posterior aspect of the apex patellae to the most superior point of the tibial tuberosity (reflecting patellar tendon length), total patellar length, patellar articular surface length, and the distance between the distal end of the patellar articular surface and the anterior edge of the tibia—measured both perpendicular to a tangent line of the medial tibia plateau. These measurements were used to calculate the CDI, ISI and BPI. Each index has established cutoff values to distinguish normal patellar height from patella alta or patella baja [9, 11, 25, 37] (Table 1).

**Table 1:** Common patella height indices in conventional lateral knee X‐rays as reported in the original publications.

Index	Calculation	Baja (infera)	Normal range	Alta
Caton–Deschamps	Ratio of the distance between the lower edge of the articular surface of the patella from the anterosuperior angle of the tibial plateau and the length of the articular surface of the patella	<0.6	0.6−1.2	>1.2^a^
Insall–Salvati	Ratio of the distance from posterior aspect of the apex patellae to the most superior point of the tibial tuberosity (patellar tendon length) to the patellar length	<0.8	0.8−1.2	>1.2
Blackburne–Peel	Ratio of the distance of an anteriorly extended line of the tibial plateau perpendicular to the lower edge of the articular surface of the patella and the length of the patellar articular surface 0.8−1.0	<0.54	0.54−1.06	>1.06

^a^ Several authors use a cut‐off >1.3l.

### Treatments

Anatomic MPFL reconstruction was performed using a doubled gracilis tendon graft passed through two transpatellar tunnels [20, 39]. With the knee in 30° of flexion, the graft was secured in two 4.0 mm patellar sockets (the proximal 3.0 mm distal to the medioproximal corner; the distal 15–20 mm inferiorly) using 4.75 mm SwiveLock™ anchors (Arthrex). Following fluoroscopic localisation of Schoettle's point, a 5.0 mm femoral tunnel was drilled, and the graft was fixed with a 6 × 25 mm bio‐tenodesis screw [45].

TTO was performed as a medializing and distalizing (multiplanar) sliding osteotomy [27, 41, 54] using an oscillating saw in the coronal plane. The resulting tuberosity fragment, at least 6 cm in length, was harvested with subcortical thickness and completed via a distal transverse cut. Simultaneous medialization and distalization were executed to achieve tibial tuberosity–trochlear groove (TT–TG) distances *of 15 mm or less and CDI of 1.0* based on preoperative planning. Internal fixation was achieved using two 3.5 mm small‐fragment lag screws to ensure interfragmentary compression; no plates were utilised in any case. Intraoperatively, patellar tracking was routinely assessed following provisional fixation of the tuberosity fragment, with the final position optimised as necessary prior to definitive stabilisation. Bereiter trochleoplasty was performed in patients with high‐grade trochlear dysplasia (Dejour types C, and D), following the surgical protocol described by Wurm et al. [33, 56].

### Data analysis

Descriptive statistics included the number of cases, mean values and standard deviations (SDs) of radiographic indices. Continuous variables were presented as mean ± SD or median with range, depending on data distribution. Confidence intervals (95% CIs) were reported where applicable. Normality was assessed using the Shapiro–Wilk test, and variance homogeneity was verified with the Levene test. Post‐ versus preoperative differences were analysed via pairwise comparisons. Group differences were evaluated using independent *t*‐tests or one‐way analysis of variance (ANOVA) (with Student‐Newman–Keuls post‐hoc tests, *α* = 0.05).

A two‐tailed one‐sample *t*‐test (test value = 0) was used to assess whether these observed changes differed significantly from zero, accounting for the minority of cases with postoperative height increases Cohen's *d* quantified effect sizes [12]. Pearson correlations assessed relationships between indices, while Bland–Altman analyses evaluated agreement [10]. Classification of concordance (patella baja/normal/alta) was analysed using linear weighted Cohen's kappa [12]. Differences in group frequencies were assessed using either Fisher's exact test or the Freeman–Halton extension of Fisher's exact test, as appropriate. All analyses were performed in MedCalc (v23.0.6, MedCalc Software Ltd).

## RESULTS

### Screening and inclusion (study population)

Between 2010 and 2022, 104 patients who underwent patellofemoral stabilisation surgery at the Department of Orthopaedics and Trauma Surgery, Medical University of Innsbruck, were screened. A total of 73 knees from 71 patients met the inclusion criteria, including 37 right knees. Reasons for exclusion were prior or concomitant axial realignment osteotomies (*n* = 6), missing or non‐evaluable radiographs (*n* = 20), and other reasons (*n* = 7).

The study population had a median age of 28 years (interquartile range: 23–33), with 23 male and 48 female participants. All knees (*n* = 73) underwent MPFL reconstruction, with 41 procedures combined with trochleoplasty, 22 with TTO, and 10 with both trochleoplasty and TTO. In total, 32 knees received TTO, while 41 did not. The median time interval between surgery and the postoperative radiograph was 176 days (95% CI: 77–254; range: 4–2349 days).

### Patella height indices

Preoperatively, the mean values of all three patellar height indices fell within established normal ranges, with the ISI approaching the upper limit of normal (1.2). Differences between preoperative and postoperative index values were normally distributed across all surgical subgroups, as confirmed by Shapiro–Wilk *p*‐values > 0.7. Overall, patellofemoral stabilisation surgery led to a statistically significant reduction in patellar height across all three indices (Table 2).

**Table 2:** Means and standard deviations of the three indices studied in 73 knees who underwent patellofemoral stabilisation surgery.

Index	Preoperative	Postoperative	Difference	In percent of preop value	*p*
Caton–Deschamps	1.1 ± 0.21	0.9 ± 0.18	−0.16 ± 0.197	−12.6 (−16.9 to −8.4)	<0.001
Insall–Salvati	1.2 ± 0.25	1.1 ± 0.22	−0.13 ± 0.171	−10.2 (−13.3 to −7.1)	<0.001
Blackburne–Peel	0.9 ± 0.2	0.8 ± 0.19	−0.15 ± 0.207	−14.1 (−19.2 to −8.9)	<0.001

*Note*: Data show pre‐ and post‐operative values and perioperative differences. *p*‐values: paired samples *t*‐test.

Comparison of pre‐ to postoperative changes, stratified by surgical technique, revealed significant differences among the three surgical groups (all ANOVA *p* < 0.05). The detailed numerical results for each index, categorised by type of surgery, are provided in Table 3.

**Table 3:** Pre‐ to postoperative changes in the three evaluated patellar height indices, stratified by surgical technique (each combined with MPFL reconstruction), followed a normal distribution (all Shapiro–Wilk *p* > 0.7) and demonstrated homogenous variances (all Levene *p* > 0.26).

Index	Trochleoplasty		TTO		Trochleoplasty +TTO		
	*n*	Mean ± SD	*n*	Mean ± SD	*n*	Mean ± SD	*p*
Caton–Deschamps	41	−0.096 ± 0.176	22	−0.2 ± 0.185	10	−0.3 ± 0.219	0.005
Insall–Salvati	41	−0.091 ± 0.157	22	−0.21 ± 0.183	10	−0.15 ± 0.154	0.026
Blackburne–Peel	41	−0.085 ± 0.188	22	−0.22 ± 0.176	10	−0.28 ± 0.252	0.004

*Note*: Reported *p*‐values reflect results from ANOVA. Abbreviations: ANOVA, analysis of variance; MPFLR, medial patellofemoral ligament reconstruction; TTO, tibial tubercle osteotomy.

**Figure 1:**
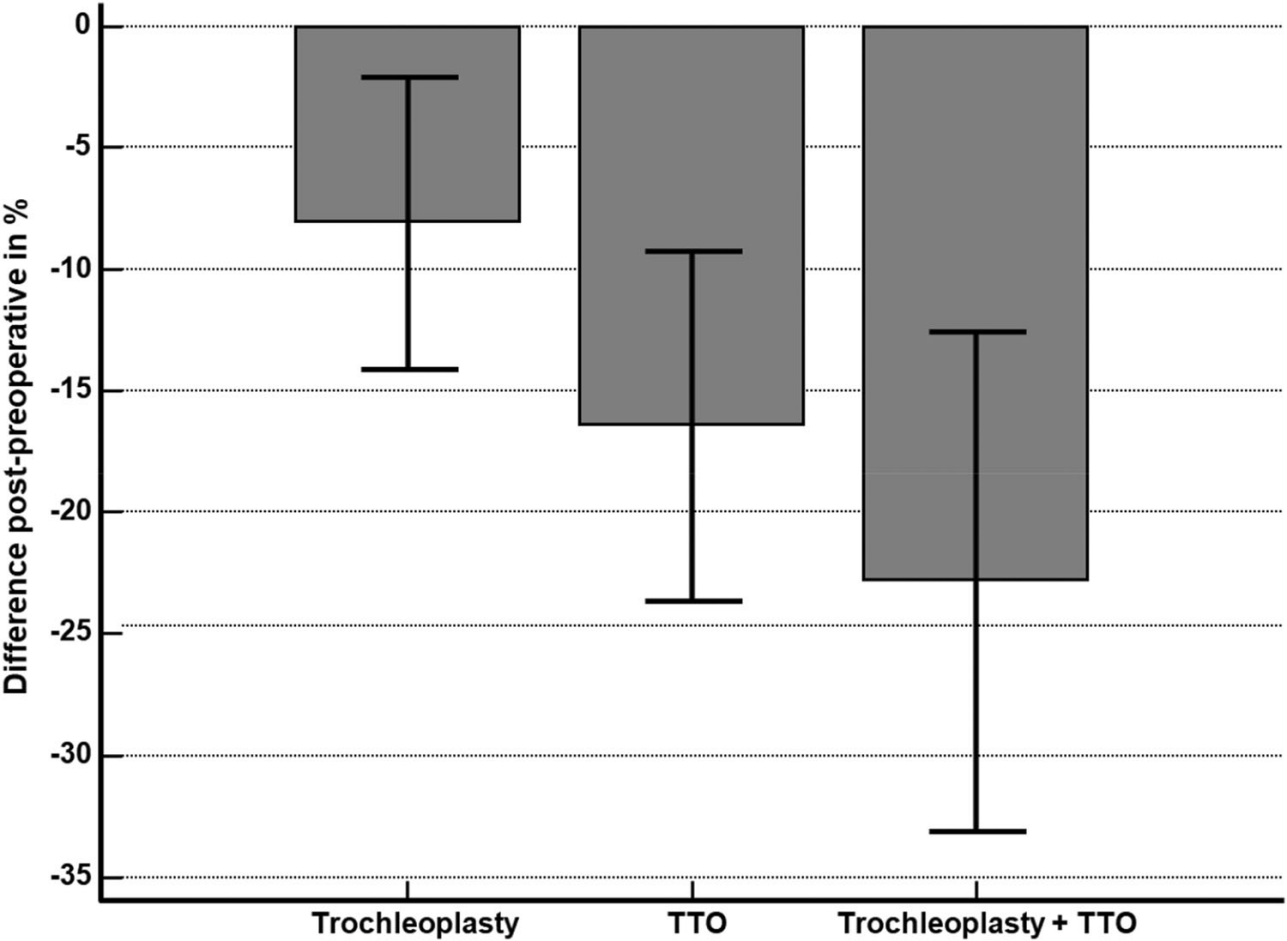
Percent reduction in patellar height as measured by the Caton–Deschamps index, stratified by surgical technique: medial patellofemoral ligament reconstruction (MPFLR) + trochleoplasty (*n* = 41), MPFLR + tibial tuberosity osteotomy (TTO; *n* = 22) and combined trochleoplasty with TTO (*n* = 10). All procedures were performed concurrently with MPFLR.

The percentage change from preoperative to postoperative values for the CDI is shown in Figure 1. One‐sample *t*‐tests revealed that trochleoplasty combined with MPFL reconstruction led to a significant reduction in the CDI by approximately −8.1% (95% CI: −14.1% to −2.1%; *p* < 0.001) of the preoperative value. TTO with MPFL reconstruction resulted in a reduction of −16.5% (95% CI: −23.7% to −9.3%; *p* < 0.05 compared to MPFL reconstruction + trochleoplasty), while the combination of trochleoplasty and TTO produced the largest decrease at −22.9% (95% CI: −33.1% to −12.6%; *p* < 0.05 compared to MPFLR + TTO).

### Patella alta conversion frequencies

Preoperatively, patella alta was present in 21 of 73 knees (28.8%) according to the CDI. In the MPFLR + trochleoplasty group, all seven knees with preoperative patella alta were corrected to a normal patellar height. In the MPFL + TTO group, five of eight knees (62.5%) converted to normal, while three remained in the alta category. In the combined group (MPFLR, trochleoplasty and TTO), five of six knees (83.3%) shifted below the Alta threshold. The Freeman–Halton test revealed no significant difference in the relative frequency of patella alta reduction between the three surgical techniques (*p* = 0.44).

### MPFL reconstruction and trochleoplasty alone lowers patella height

As the primary objective of this study, the absolute magnitude of patellar height reduction was evaluated to quantify the isolated effect of combined MPFL reconstruction and trochleoplasty without TTO (*n* = 41). Effect sizes were quantified using Cohen's *d*, with detailed results for all three indices summarised in Table 4.

**Table 4:** Perioperative changes in patellar height indices (*n* = 41).

Index	Mean (95% confidence interval)	*p*	Cohen's *d*
Caton–Deschamps	−0.096 (−0.152 to −0.0404)	0.0012	0.55
Insal–Salvati	−0.091 (−0.14 to −0.041)	<0.001	0.61
Blackburne–Peel	−0.0854 (−0.145 to −0.0260)	0.006	0.45

*Note*: The data compare preoperative and postoperative measurements in patients undergoing MPFL reconstruction and trochleoplasty without TTO. *p*‐Values are based on one‐sample *t*‐tests comparing observed differences to a null hypothesis of zero change. Effect sizes, calculated as Cohen's *d*, indicate medium‐sized effects.

MPFLR and trochleoplasty alone resulted in an average reduction of approximately 0.09 across all three indices, equivalent to a 10% decrease from baseline. While the absolute reduction was modest, all changes were significant and demonstrated medium effect sizes. Notably, the reduction achieved without TTO was about 40% of that observed in patients who underwent TTO. Compared to the TTO group, the CDI, ISI and BPI decreased by 41%, 48% and 36%, respectively.

### Temporal distribution of patellar height changes

**Figure 2:**
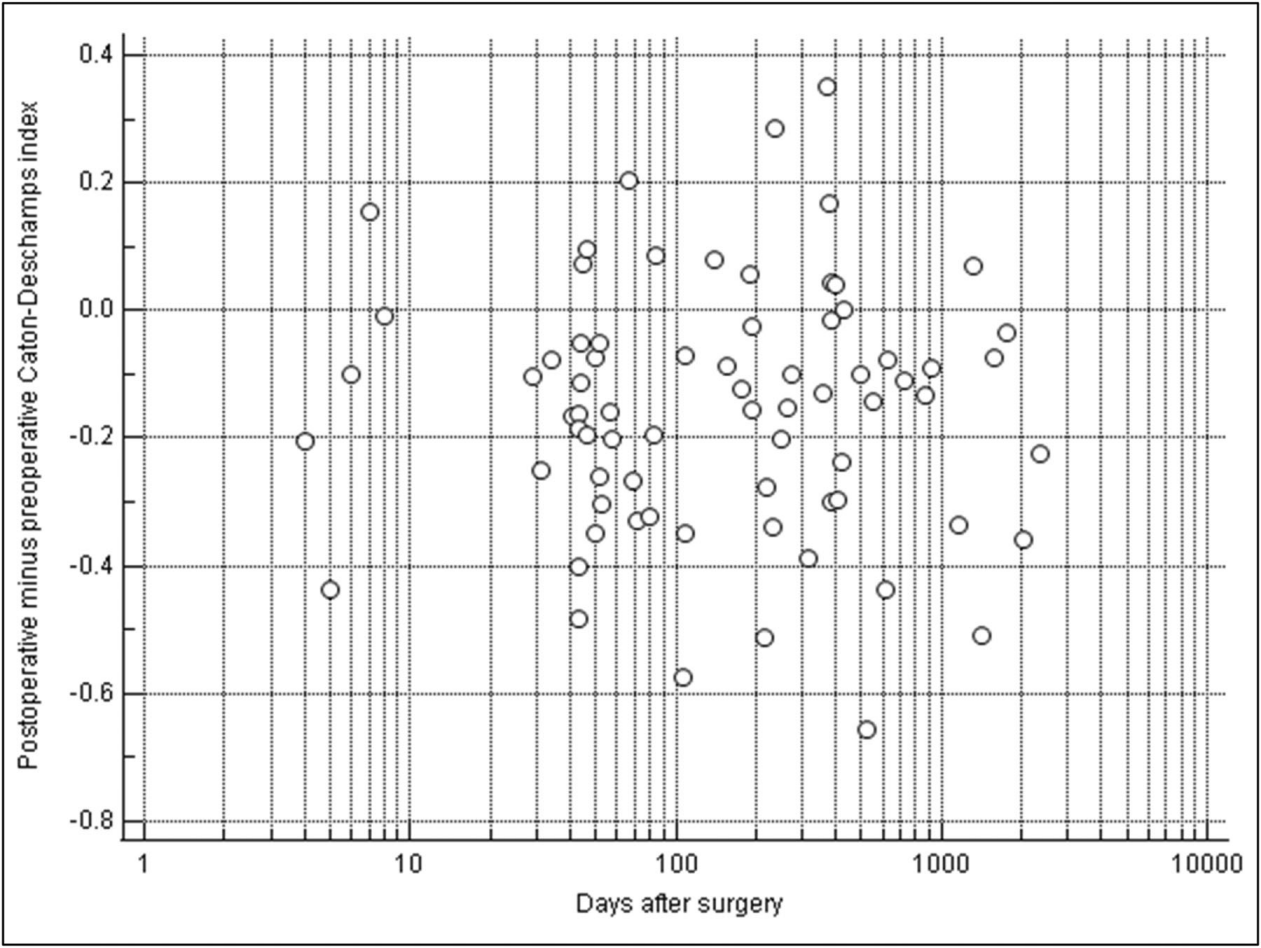
Temporal distribution of patellar height changes of the Caton–Deschamps index following patellofemoral stabilisation surgery. The plot shows the immediate postoperative reduction in the Caton–Deschamps index with no significant subsequent changes. Linear regression analyses of the Insall–Salvati and Blackburne–Peel indices also revealed initial reductions and index stability over time.

Linear regression was performed to analyse the relationship between the postoperative‐to‐preoperative index differences and the respective time of follow‐up for each patient. The analysis demonstrated a significant immediate postoperative reduction in patellar height (CDI intercept: −0.15; ISI: −0.14; BPI: −0.16; all *p* < 0.0001). No significant correlation was found between the magnitude of reduction and the duration of follow‐up (slope: −3.0 × 10^−5^/day for CDI, *p* = 0.56; +1.5 × 10^−5^/day for ISI, *p* = 0.73; 8.0 × 10^−6^/day for BPI, *p* = 0.88). These findings indicate that the surgically achieved reduction in patellar height remained consistent across the varied postoperative intervals represented in the study population (Figure 2).

### Changes in patella–tibia distance and patellar length measurements

All patellar height indices represent ratios of a patella–tibia distance to a longitudinal patellar dimension. The significant postoperative reductions observed across these indices appear to be primarily associated with decreased patella–tibia distances (Table 5). This is of particular interest regarding the ISI, as it directly measures the bony landmarks representing patellar tendon length. In our cohort, this distance decreased significantly from 50.0 to 45.7 mm (mean reduction: 4.26 mm; *p* < 0.0001). Although no reference ball was used to calibrate for magnification, pre‐ to post‐op length measurements of the patella were consistent (<1 mm), so these relative changes still seem to be relevant.

**Table 5:** Pre‐ to postoperative changes in patellar length and patella‐tibia distance—the numerator and denominator components of the three indices—were analysed in 73 patients following patellofemoral stabilisation surgery.

Measure of	Index	Mean difference (mm)	95% confidence interval (mm)	*p*
Patella‐tibia distance (index numerator)	Caton–Deschamps	−4.43	−5.91 to −2.94	<0.0001
	Insall–Salvati	−4.26	−5.79 to −2.72	<0.0001
	Blackburne–Peel	−4.45	−6.04 to −2.85	<0.0001
Patellar length (index denominator)	Caton–Deschamps	0.45	−0.11 to 1.01	n.s.
	Insall–Salvati	1.04	0.37 to 1.71	0.0027
	Blackburne–Peel	0.40	−0.17 to 0.97	n.s.

*Note*: The *p*‐values reflect one‐sample *t*‐tests comparing observed differences to a null hypothesis of 0 mm change. Abbreviation: n.s., not significant.

### Agreement between patellar height indices

Agreement among radiological patella height indices was evaluated using multiple metrics. Preoperative Pearson correlation coefficients (*n* = 73) ranged from 0.56 to 0.9, demonstrating strong and statistically significant linear associations (all *p* < 0.001). Specifically, correlations were 0.56 between CDI and ISI, 0.9 between CDI and BPI, and 0.58 between ISI and BPI.

**Figure 3:**
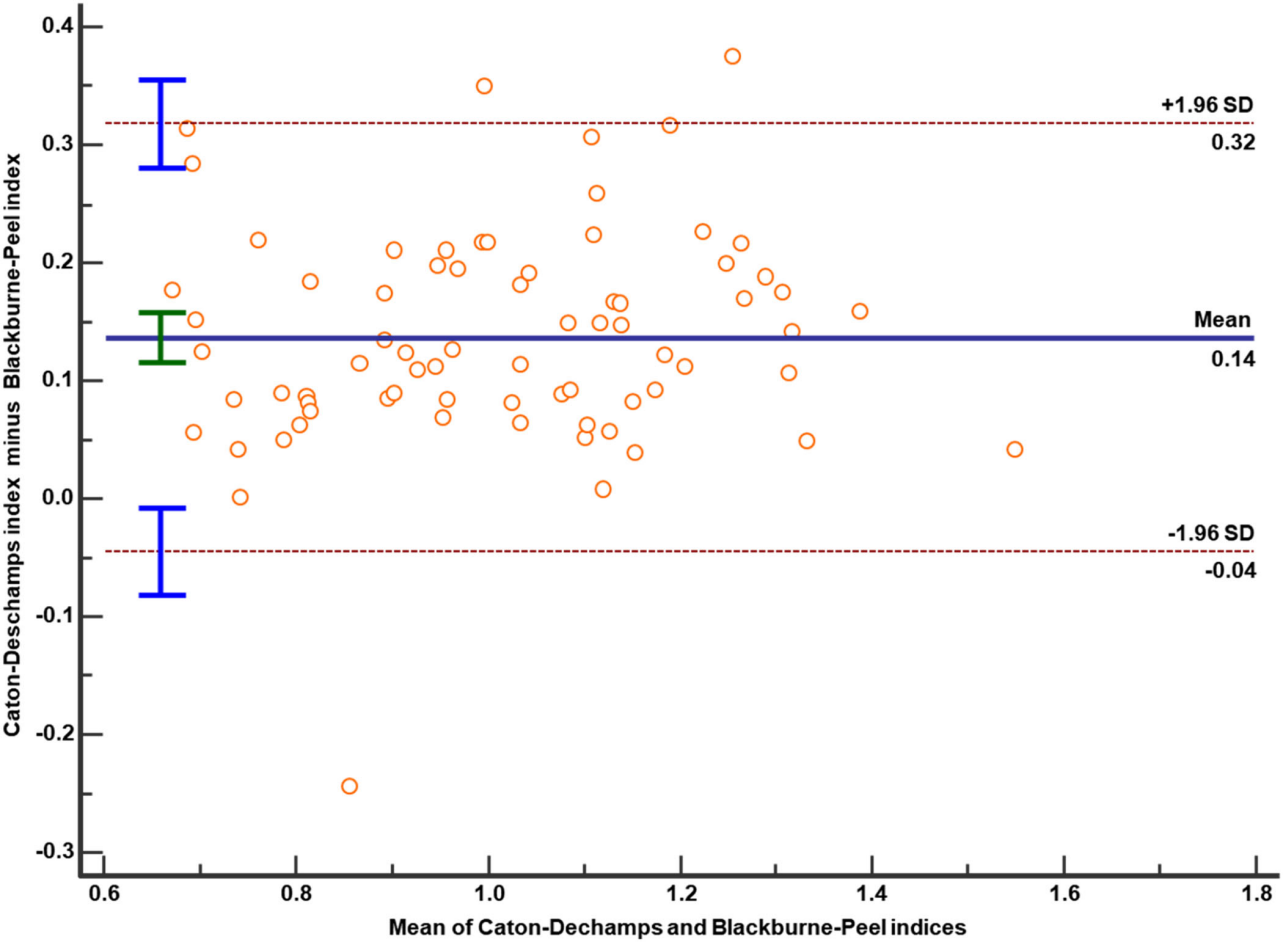
Bland–Altman plot comparing Caton–Deschamps and Blackburne–Peel indices (*n* = 73). The solid line represents the mean difference (bias), while the dashed lines indicate the 95% limits of agreement (±1.96 SD). Corresponding bars denote the 95% confidence intervals for both the bias and the limits of agreement.

Bland–Altman analysis revealed systematic differences between the indices. The BPI exceeded the CDI by a bias of 0.14 units (*p* < 0.001; Figure 3). The ISI showed a bias of −0.12 units (95% CI: −0.17 to −0.07, *p* < 0.001) compared to the CDI, with limits of agreement ranging from −0.55 to 0.30. The largest bias was observed between ISI and BPI (0.26 units, 95% CI: 0.21 to 0.32, *p* < 0.001), with limits of agreement from −0.15 to 0.67, indicating that the ISI consistently yielded higher values.

**Figure 4:**
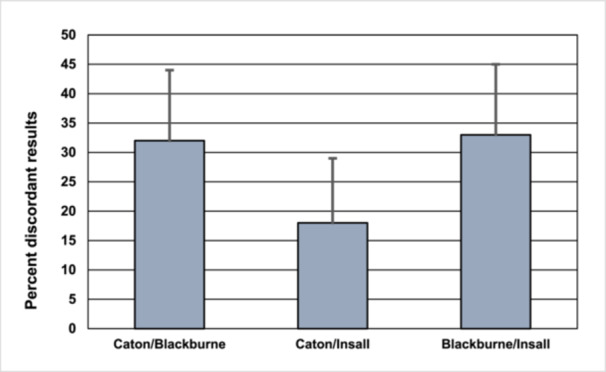
Percent discordant classifications of patella height based Caton–Deschamps, Blackburne–Peel and Insall–Salvati indices in 73 patients with patellar instability. Error bars represent 95% confidence intervals.

Preoperative classification of patellar height (alta, normal or baja) using the specific cut‐off values from the original publications (Table 1) yielded discordant results in 18%–33% of knees (Figure 4). Cohen's kappa values indicated only fair to moderate agreement between the indices: 0.37 for CDI versus ISI, 0.60 for CDI versus BPI, and 0.36 for ISI versus BPI. Exemplarily, the CDI identified 22 of 73 patellae as alta, whereas the ISI classified 34 patellae as alta (Fisher's exact *p* = 0.005).

### Iatrogenic patella baja

The potential cumulative effect of trochleoplasty and TTO on patellar height reduction prompted an evaluation of the risk of postoperative iatrogenic patella baja. The incidence of patella baja varied substantially depending on the index used (Table 1). Postoperatively, the CDI identified three knees (two after isolated trochleoplasty and one after TTO) as baja, while the ISI identified seven knees (five without TTO and two with TTO). The BPI, however, classified 38 instances of patella baja—20 without TTO and 18 with TTO.

## DISCUSSION

The MPFL provides the primary soft‐tissue restraint for patellar tracking, and its reconstruction serves as the surgical baseline procedure in PFI. In severe trochlear dysplasia, additional trochleoplasty restores the essential bony constraint as the knee moves into deeper flexion [13, 24, 44]. Patella alta impairs this transition by delaying patellar entry into the trochlear groove, leaving the joint vulnerable during the initial 30° of flexion [1, 7, 50]. Consequently, the present study evaluated three surgical combinations for patellar stabilisation: MPFL reconstruction with trochleoplasty, MPFL reconstruction with TTO, and a triple procedure comprising MPFL reconstruction, trochleoplasty and TTO.

Radiographic indices—including the CDI, ISI and BPI—are essential for diagnosing patella alta and guiding the indication for distalizing TTO [1, 23, 24, 34, 38]. In clinical practice, these scalar indices are used to classify patellar height as patella baja, normal height, or patella alta. When patella alta is diagnosed—defined by index values exceeding rigid cut‐off thresholds—a distalizing TTO is frequently indicated, eventually combined with medializing TTO [1, 7, 14, 34, 50]. As cut‐off thresholds for classifying patellar height vary inconsistently across the literature [1, 8, 22, 46], the present study applied the specific cut‐off values defined in their respective original publications [9, 11, 25].

### Trochleoplasty with MPFL reconstruction lowers the patella without TTO

This study investigated whether combined MPFL reconstruction and trochleoplasty can effectively reduce patellar height without TTO. The findings suggest that combined trochleoplasty and MPFL reconstruction alone significantly lowers patellar height across all three indices, achieving approximately 40% of the reduction typically observed with TTO. This corresponds to a medium effect size [12] and aligns with earlier reports [4, 28], indicating that TTO may be avoidable in selected patients with patella alta based on radiographic indices. The lack of significant differences in patella height conversion rates between the surgical groups (*p* = 0.44) suggests that isolated MPFL reconstruction and trochleoplasty achieve a distalizing effect similar to that of procedures combined with TTO. However, these categorical findings should be viewed as supplementary to the more sensitive results obtained from continuous index measurements.

Hunter et al. observed that patellar height, as measured by the CDI, tended to return towards preoperative values over time after MPFL reconstruction, with or without TTO [21]. Specifically, MPFL alone initially decreased patellar height in their study, which then progressively increased, whereas MPFL combined with TTO showed minimal changes overall. To determine whether the postoperative index changes were independent of the respective follow‐up intervals, a linear regression was performed. This analysis confirmed that the measured distalization was not associated with the duration between surgery and postoperative radiograph (all *p* ≥ 0.56). Regression analysis indicated an immediate postoperative distalization that remained consistent across the varied follow‐up intervals (Figure 2). Although direct time trajectories were not modelled, the consistency of indices across follow‐up intervals suggests that patellar height remained stable postoperatively.

The reductions in patellar height from trochleoplasty and TTO were cumulative (Figure 1). However, no cases of patella baja were observed according to the CDI postoperatively. Analysis of postoperative ISI and BPI values suggests that while iatrogenic patella baja can occur following patellofemoral stabilisation, its incidence does not appear to correlate with the specific surgical technique employed. Furthermore, current evidence indicates no increased risk of patella baja through unintentional overcorrection in combined procedures [29].

### Structures involved in the decrease of patellar height

Although a secondary finding, all three indices demonstrated a consistent, immediate and stable postoperative reduction in the radiographic index numerator, representing the patella–tibia distance (Table 5). The consistent decrease of approximately 4 mm across all surgical groups suggests a systematic effect attributable to MPFL reconstruction, which—in accordance with current guidelines [23, 38]—represented the only common procedural element among all cohorts. Notably, specific techniques such as patellar tunnel placement and graft anchoring have been reported to reduce patellar height in isolated MPFL reconstruction [19, 28, 47, 55], though findings on this effect remain inconsistent [17, 26, 42]. Since the patellar tendon was not surgically addressed, the exact mechanism for this systematic reduction of the patella–tibia distance remains unclear. It may reflect scarification of surrounding soft tissues, a 3D reorientation of the patella (‘nosing‐down’ effect) affecting 2D radiographic projections, or subtle shifts in identifiable bony reference points following joint stabilisation. The low variability of this finding suggests a consistent biomechanical response to patellofemoral surgery that warrants further investigation. *The significant change of patellar length in the ISI is attributed to changes in the radiographic projection of the patellar apex* [46].

### Concerns about validity of patella height cut‐off thresholds

Identifying patella alta in patients with PFI carries significant clinical implications, highlighting the need for a critical evaluation of radiographic indices and their cut‐off thresholds. While correlations among indices may reflect construct validity—with strong preoperative Pearson coefficients—Bland‐Altman analyses revealed systematic biases of 10%–25% of mean values.

In clinical practice, patellar height is categorised into baja, normal, or alta based on threshold values, often guiding decisions for TTO. However, index‐based classifications proved inconsistent in up to a third of the knees (Figure 4), with Cohen's kappa values between 0.36 and 0.6. Preoperatively, patella alta was diagnosed in 22 patients using the CDI, 22 with the BPI, and 34 with the ISI. These discrepancies raise concerns about the clinical validity of proposed cut‐off thresholds, an issue echoed in recent literature. Given the inconsistent cut‐off thresholds reported in secondary literature [1, 8, 22, 46, 48], the original thresholds defined in the primary publications were utilised for this study [9, 11, 25]. Alternative cut‐off thresholds proposed in the literature were evaluated comparatively but did not yield superior concordance rates (data not shown).

Among the three indices, the CDI was conservative in recommending patella‐lowering surgery and is favoured by many experts [23, 24, 38]. Palmowski et al. demonstrated good agreement between this index and MRI‐based parameters, such as the patellotrochlear index in patients with normal TT‐TG distances [35], although inconsistencies have also been reported [48]. Luceri et al. suggested that bony procedures may be avoided in patients with patellar instability if the CDI is up to 1.4 [31].

Recent publications increasingly emphasise the use of additional MRI‐based measurements for patella‐lowering surgery [38, 40, 49, 53], and current AOSSM guidelines recommend incorporating MRI‐based criteria for surgical indications for TTO [38]. The findings of this study further challenge the suitability of relying solely on rigid radiological thresholds to indicate TTO, especially given the concurrent effects of additional surgical interventions on patellar height. The potential role of AI‐based assessment methods in future clinical practice remains to be explored [5].

### Limitations

Potential selection bias and limited representativeness are limitations of this study. Of 104 screened adults, 71 (73 knees) were enrolled; exclusions were primarily due to the legal exclusion of minors (<18 years) and the requirement for complete pre‐ and postoperative imaging. While these criteria narrowed the cohort, they ensured a complete and systematically evaluated dataset. The screening process is reported transparently to account for these limitations.

The variable follow‐up intervals resulted from prioritising radiographic quality; suboptimal scans with rotation or flexion errors were replaced by the nearest available images of adequate quality. Linear regression indicates that index changes remained independent of follow‐up duration (Figure 2).

Although the inclusion of two bilateral cases (*n* = 73 knees/71 patients) introduces a minor statistical dependency (2.7\%), sensitivity analysis comparing the full cohort with a strictly independent subset (*n* = 71) yielded identical results to the second decimal place. As this correlation did not impact clinical or statistical conclusions, all cases were retained for maximum transparency.

Due to the combined medialization and distalization in multiplanar TTO, the specific frequency and precise magnitude of medialization or distalization performed in each individual knee were not recorded.

## CONCLUSION

Trochleoplasty combined with MPFL reconstruction results in a significant reduction in patellar height. This systematic interaction effect of various PFI procedures, coupled with methodological inconsistencies in index‐based classification, challenges the validity of rigid cut‐off values for indicating distalizing TTO. Among the three indices evaluated, the ISI (cutoff > 1.2) was the most progressive in diagnosing patella alta.

## AUTHOR CONTRIBUTIONS

All authors contributed to the study conception and design. Material preparation, data collection and analysis were performed by Felix Riechelmann, Nadja Gasser, Alexa Schaufler, Wolfgang Hackl and Rohit Arora. The first draft of the manuscript was written by Felix Riechelmann and all authors commented on previous versions of the manuscript. All authors read and approved the final manuscript.

## CONFLICT OF INTEREST STATEMENT

The authors declare no conflicts of interest.

## ETHICS STATEMENT

The study protocol was approved by the Ethics Committee of the Medical University of Innsbruck (Ethics Approval No.: 1316/2022). In view of the retrospective nature of the study and all the procedures being performed were part of the routine care.

## Data Availability

The data that support the findings of this study are available from the corresponding author upon reasonable request.
